# Sleep and Alcohol Use Patterns During Federal Holidays and Daylight Saving Time Transitions in the United States

**DOI:** 10.3389/fphys.2022.884154

**Published:** 2022-07-11

**Authors:** Rachel M. Heacock, Emily R. Capodilupo, Mark É. Czeisler, Matthew D. Weaver, Charles A. Czeisler, Mark E. Howard, Shantha M. W. Rajaratnam

**Affiliations:** ^1^ Whoop Inc., Boston, MA, United States; ^2^ Turner Institute for Brain and Mental Health and School of Psychological Sciences, Monash University, Melbourne, VIC, Australia; ^3^ Institute for Breathing and Sleep, Austin Health, Heidelberg, VIC, Australia; ^4^ Department of Psychiatry, Brigham and Women’s Hospital, Boston, MA, United States; ^5^ Francis Weld Peabody Society, Harvard Medical School, Boston, MA, United States; ^6^ Division of Sleep and Circadian Disorders, Departments of Medicine and Neurology, Brigham and Women’s Hospital, Boston, MA, United States; ^7^ Division of Sleep Medicine, Harvard Medical School, Boston, MA, United States; ^8^ Department of Medicine, University of Melbourne, Melbourne, VIC, Australia

**Keywords:** sleep timing, sleep consistency, sleep duration, substance use, holidays, daylight saving time, wearable devices, epidemiology

## Abstract

We conducted a retrospective observational study using remote wearable and mobile application data to evaluate whether US public holidays or Daylight Saving Time transitions were associated with significant changes in sleep behaviors, including sleep duration, sleep onset and offset, and the consistency of sleep timing, as well as changes in the point prevalence of alcohol use. These metrics were analyzed using objective, high resolution sleep-wake data (10,350,760 sleep episodes) and 5,777,008 survey responses of 24,250 US subscribers (74.5% male; mean age of 37.6 ± 9.8 years) to the wrist-worn biometric device platform, WHOOP (Boston, Massachusetts, United States), who were active users during 1 May 2020, through 1 May 2021. Compared to baseline, statistically significant differences in sleep and alcohol measures were found on most DST transitions, US public holidays, and their eves. For example, New Year’s Eve corresponded with a sleep consistency decrease of 13.8 ± 0.3%, a sleep onset delay of 88.9 ± 3.2 min (00:01 vs*.* 22:33 baseline) later, a sleep offset delay of 78.1 ± 3.1 min (07:56 vs*.* 06:39), and an increase in the prevalence of alcohol consumption, with more than twice as many participants having reported alcohol consumption [+138.0% ± 6.7 (74.2% vs*.* 31.2%)] compared to baseline. In this analysis of a non-random sample of mostly male subscribers conducted during the COVID-19 pandemic, the majority of US public holidays and holiday eves were associated with sample-level increases in sleep duration, decreases in sleep consistency, later sleep onset and offset, and increases in the prevalence of alcohol consumption. Future work would be warranted to explore the generalizability of these findings and their public health implications, including in more representative samples and over longer time intervals.

## Introduction

In the United States, the government and workplaces observe 10 public holidays annually. In 2020 and 2021, each holiday corresponded to approximately 151 million employed persons receiving a day off from work (U.S. Bureau of Labor Statistics, 2021). Changes to sleep associated with time off from work have predominantly been investigated in the context of vacations (Westman and Etzion, 2001; de Bloom et al., 2013) and not specific holidays, while studies of alcohol use patterns have found that consumption of alcohol among regular drinkers increases on holidays (Kushnir and Cunningham, 2014) and is substantially elevated on special occasions (Bellis et al., 2015). Daylight Saving Time (DST) transitions also represent discrete events altering regular scheduling due to the resetting of clocks. The transition of moving clocks forward by 1 h for DST in the spring reportedly contributes to shortened sleep and is associated with increased motor vehicle crashes, workplace injuries, heart attacks, and all-cause mortality (Barnes and Wagner, 2009; Sandhu et al., 2014; Manfredini et al., 2019; Fritz et al., 2020; Poteser and Moshammer, 2020). However, changes to both sleep and alcohol use on specific holidays over the course of a year have not been comprehensively characterized. As such, we sought to identify US public holidays and DST transitions associated with significant changes in health behaviors, including sleep duration, consistency, and timing, as well as alcohol use.

Weekly changes in sleep patterns can have a significant impact on overall health. The majority of working individuals change their sleep timing and duration depending on next-day commitments (Roenneberg and Merrow, 2016); this behavior results in cyclical population-level data patterns in which weekend sleep episodes are longer than weekday sleep episodes. Weekday sleep episodes are frequently of insufficient duration, such that many people accrue sleep debt during the work week, and attempt to compensate by extending the duration of weekend sleep episodes (Capodilupo and Miller, 2021). Differences in the timing of sleep on free days and work days can result in a misalignment between an individual’s circadian rhythms and the timing of social constraints (i.e., social jetlag) (Wittmann et al., 2006). In these cases, social jetlag results in weekly population-level changes in sleep behavior in which weekend sleep duration is longer and the timing of sleep onset and sleep offset occur later compared to weekday sleep duration and timing. Such increased variability in sleep habits is associated with obesity, two-fold increased risk of metabolic syndrome, and diabetes or prediabetes, among other adverse health consequences (Roenneberg et al., 2012; Koopman et al., 2017). Conversely, past research has indicated that longer catch-up sleep episodes on weekends or free-days are sometimes beneficial, particularly among individuals who obtain insufficient sleep on workdays. Compensatory weekend catch-up sleep has been associated with a lower body mass index (BMI) (Im et al., 2017) and lower levels of high-sensitivity C-reactive protein (hsCRP) (Jung et al., 2019) in such populations. Consistent short sleep duration is associated with an increased risk of mortality, but prospective cohort studies have found that those who compensate for shorter sleep episodes during the week with longer sleep duration over the weekend do not have an increased risk of mortality (Åkerstedt et al., 2019). Like weekends, holiday eves that fall on weekdays can provide a longer window of sleep opportunity and greater ability to self-select sleep timing in the absence of work.

As with sleep, weekly differences in alcohol use patterns have been reported, with more consumption on weekends compared to weekdays (Kushnir and Cunningham, 2014; Lau-Barraco et al., 2016). Such increases have been temporally associated with increased occurrence of motor vehicle accidents among young Swiss men, with parallel peaks in alcohol consumption and alcohol-related motor vehicle accidents on Fridays and Saturdays, as well as an increase on public holidays and the prior night (Foster et al., 2015). Similarly, motor vehicle crashes in the US peak on Independence Day (4 July), and a high portion of alcohol-related crashes and elevated alcohol impairment-related pedestrian crash deaths occur on New Year’s Day (1 January) (Farmer and Williams, 2005). Moreover, the combination of legal low-dose alcohol and altered sleep (prolonged wake or sleep restriction) are synergistic, resulting in worse impairment than observed at higher alcohol levels that have been shown to increase crash risk (Philip et al., 2001; Howard et al., 2007). More broadly, alcohol use, particularly heavy alcohol consumption, is a risk factor for myriad adverse health consequences, including infectious diseases, diabetes, mental health and substance use conditions, liver and pancreatic diseases, and serious injuries (Rehm, 2011). Understanding population-level changes in alcohol use patterns may therefore inform public health efforts to reduce the occurrence of driving under the influence or operating while impaired.

This analysis aimed to investigate the magnitude of changes to sleep duration, consistency, timing, and the point prevalence of alcohol consumption on and prior to public holidays and on DST transitions in the US.

## Materials and Methods

### Data Source and Study Design

To analyze sleep and alcohol use patterns before and during federal holidays and during DST transitions, data collected from adult subscribers to the wearable biometric device platform WHOOP, Inc. (Boston, Massachusetts, United States) were analyzed retrospectively. During the 1-year study interval (1 May 2020 to 1 May 2021), WHOOP users recorded sleep-wake data *via* continuous monitoring from the WHOOP device, with optional daily surveys on health behaviors including alcohol use. With WHOOP membership growing rapidly, the study interval was chosen to optimize the balance of a growing participant pool with sufficient historical data (i.e., a calendar year). The age range chosen represents working-age individuals, who were most likely to have their work schedules impacted by public holidays (United States Census Bureau, 2021). Participants were chosen from only the US to avoid confounding the data with international differences in holiday observances. Participants without sleep-wake data on any of the public holidays were excluded from the analysis. Additionally, individual sleep episodes for which complete data were not available (for example, due to loss of device charge) were not counted towards participants’ required minimum of 70% compliance for inclusion.

Inclusion criteria for the primary analytic sample (sleep data) were: 1) being a US-based member of the WHOOP platform who collected data for 255 (70%) or more days out of the year-long study interval from 1 May 2020 through 1 May 2021; and 2) being between the ages of 18 and 65 years for the entirety of the data collection interval. From the primary analytic sample, a subsample of participants who responded to optional daily surveys about alcohol consumption for 255 (70%) or more days and all holidays during the data collection interval were included in the alcohol use subsample.

Holidays analyzed included all 10 US public holidays as observed by the Federal Reserve System (Federal Reserve Bank of Chicago, 2021): Memorial Day (25 May 2020), Independence Day (also known as Fourth of July; 4 July 2020), Labor Day (7 September 2020), Columbus Day (also known as Indigenous Peoples’ Day or Indigenous Peoples Day; 12 October 2020), Veterans Day (11 November 2020), Thanksgiving Day (26 November 2020), Christmas Day (25 December 2020), New Year’s Day (1 January 2021), Martin Luther King Jr Day (also known as Martin Luther King Junior’s Birthday; 18 January 2021), and Presidents’ Day (also known as Washington’s Birthday; 15 February 2021). DST transitions included setting clocks earlier 1 h in the fall (“Fall Back”; 1 November 2020) and setting clocks later by 1 h in the spring (“Spring Ahead”; 14 March 2021).

The Monash University Human Research Ethics Committee reviewed and approved the study protocol (#26304). All participants in the study consented at registration with WHOOP to the use of their data for the purposes of scientific research and could withdraw consent at any time.

### Outcome Variables

Sleep data were derived from data captured using a wrist-worn multi-sensor (tri-axial accelerometer, optical heart rate sensor, capacitive touch sensor and ambient temperature sensor) on the WHOOP device (Berryhill et al., 2020). Sleep variables included in this analysis were sleep duration, sleep onset, sleep offset, and the consistency of sleep timing. Sleep consistency was measure using a proprietary method adapted from the previously validated Sleep Regularity Index (SRI) (Phillips et al., 2017), which is the percentage of concordance of individuals being in the same state (asleep versus awake) at different timepoints. Whereas the SRI compares two total timepoints 24 h apart, the WHOOP sleep consistency compares 24-h timepoints over a 4-day interval (Czeisler et al., 2022). Additional timepoints were included to incorporate additional recent historical sleep-wake data into the WHOOP sleep consistency score, with the intervals assigned progressively lower weights as they become further apart. Four sleep outcomes were included given increased recognition of the multidimensionality of sleep (Buysse, 2014).

The WHOOP device has undergone multiple performance assessment in young, healthy individuals (Berryhill et al., 2020; Miller et al., 2020, Miller et al., 2021) and has demonstrated acceptable two-stage (wake or sleep) categorization to automatically detect sleep, with 86%–89% agreement with polysomnography (Miller et al., 2020; Miller et al., 2021).

For the alcohol use variable, subscribers were prompted in optional daily surveys delivered through the WHOOP mobile application, which included a question on alcohol use during the previous day, reported as a binary (yes/no).

### Statistical Analysis

First, for each holiday, the day preceding the holiday, and DST transition, the percent change to each sleep outcome relative to a baseline was calculated on the primary analytic sample, as was the percent change in the point prevalence of alcohol use within the subsample of eligible participants. To determine a baseline for holiday and DST transition comparisons, sleep and alcohol data from the same day of the week for 4 weeks preceding and 4 weeks following the date of each public holiday were averaged. For example, data for Thanksgiving Day (Thursday, 26 November 2020) were compared to data from the four calendar Thursdays preceding and four following Thanksgiving Day. The baselines were comprised of the same day of the week to account for population-level variance in sleep behaviors based on the day of the week (Capodilupo and Miller, 2021). A local rather than a complete-year baseline was selected to avoid the confounding effect of seasonal variation in sleep and alcohol use patterns and the effect of fluctuating severity of the coronavirus disease 2019 (COVID-19) pandemic and its associated mitigation policies (Capodilupo and Miller, 2021; Czeisler et al., 2022).

Days preceding each holiday (herein, eves) were analyzed separately to account for the potential impacts on both sleep and behavior leading into as well as occurring on a holiday. For example, we hypothesized that in anticipation of Labor Day, which occurs on a Monday, the preceding Sunday night might have higher prevalence of alcohol consumption, shifted sleep timing, and overall extended sleep relative to a typical Sunday night, but Labor Day itself may resemble a non-holiday Monday night in which sleep and alcohol patterns are indifferent from typical pre-work night patterns. Christmas Eve and Day and New Year’s Eve and Day are exactly 1 week apart and therefore a necessary exception to the described baseline methodology. These days were excluded from each others’ baseline calculations to avoid confounding the baseline with another holiday.

Daily sleep duration, consistency (scored on a 0 to 100 scale), and timing were calculated for participants over the year-long study interval. An average by day was taken across participants for each sleep metric to calculate the variance in metrics over the duration of the study. Sleep episodes were assigned to the calendar date in the local time zone in which the sleep ended. Both going to bed before and after midnight are common. Having used the date of wake avoided misattributing sleep episodes to different days based on the episodes beginning before or after midnight. For example, sleep episodes concluding the morning of 2 January 2020 were treated as if the sleep episode were initiated on date 1 January 2020. If the sleep episode initiated in the early morning hours of 2 January 2020, the episode was still assigned to 1 January 2020. The average values for each participant were compared to their individual baseline values and used to calculate percent changes for sleep duration and sleep consistency for both the public holidays and their eves. Sample estimates for percent change were created by averaging participant estimates. The 95% confidence intervals based on between-subjects variability were estimated as well.

The alcohol data were analyzed using the daily percentage of subscribers who reported alcohol consumption in the optional survey out of the total subscribers who responded to the question. Individuals included in this subset of analysis who did not respond on a given day were omitted from the sample for that day. Similar to the method of analyzing sleep data, a baseline using the same day of week from 4 weeks prior to and 4 weeks after each holiday was created by taking an average of the 8 days. The binomial proportion confidence interval was calculated for both the holidays and their baselines. The percent change from baseline was calculated for both the public holidays and their eves. The propagation of error for the percent change was computed, accounting for the correlation of measurement error and correlation of uncertainties in the group averages. Two-sided *p*-values were obtained from confidence intervals for a difference (Altman and Bland, 2011). The changes in individual measures (sleep duration, sleep consistency, sleep onset, sleep offset, and alcohol use) for each holiday and eve were then ranked among the holidays and eves in each measure from most positive to most negative change compared to baseline.

Second, to evaluate whether there was a relationship between alcohol use and changes in sleep patterns, for each holiday, eve, and DST transition, analyses of paired percent changes in alcohol use and 1) sleep duration and 2) sleep consistency were conducted in the subsample of eligible participants.

All analyses were conducted using the Python programming language version 3.6.2 (Python Software Foundation). The threshold for statistical significance was set at 0.05.

## Results

In total, 24,250 participants met the inclusion criteria and were included in the primary analytic sample [with objective sleep-wake data for an average of 321 out of 365 (87.9%) days], and 13,904 participants were included in the subsample with data on alcohol use.

Across participants, 10,350,760 sleep episodes and 5,777,008 alcohol survey responses were recorded and analyzed. Participants (n = 24,250) were on average aged 37.6 (±9.8) years. Male participants (*n* = 18,060, 74.5% of sample) had an average age of 37.9 (±9.8) years and female participants (*n* = 6,187, 25.5% of sample) had an average age of 37.0 (±9.6) years. Participants who reported sufficient information on alcohol (n = 13,904) had an average age of 37.8 (±9.7) years. Additional demographic information, including race and socioeconomic status, were not available.

Across all nights in the study interval, participant values for sleep duration, sleep onset, and sleep offset were normally distributed (Supplementary Figure S1). Consistent with the participants in Phillips et al. (2017) and as expected given a propensity of participants to have high concordance in sleep vs*.* wake states over 24 h intervals, values for sleep consistency were skewed to the left. In the subsample of participants who recorded alcohol use, the distribution of total days of use over total days reported is bimodal across the study interval.

**FIGURE 1:**
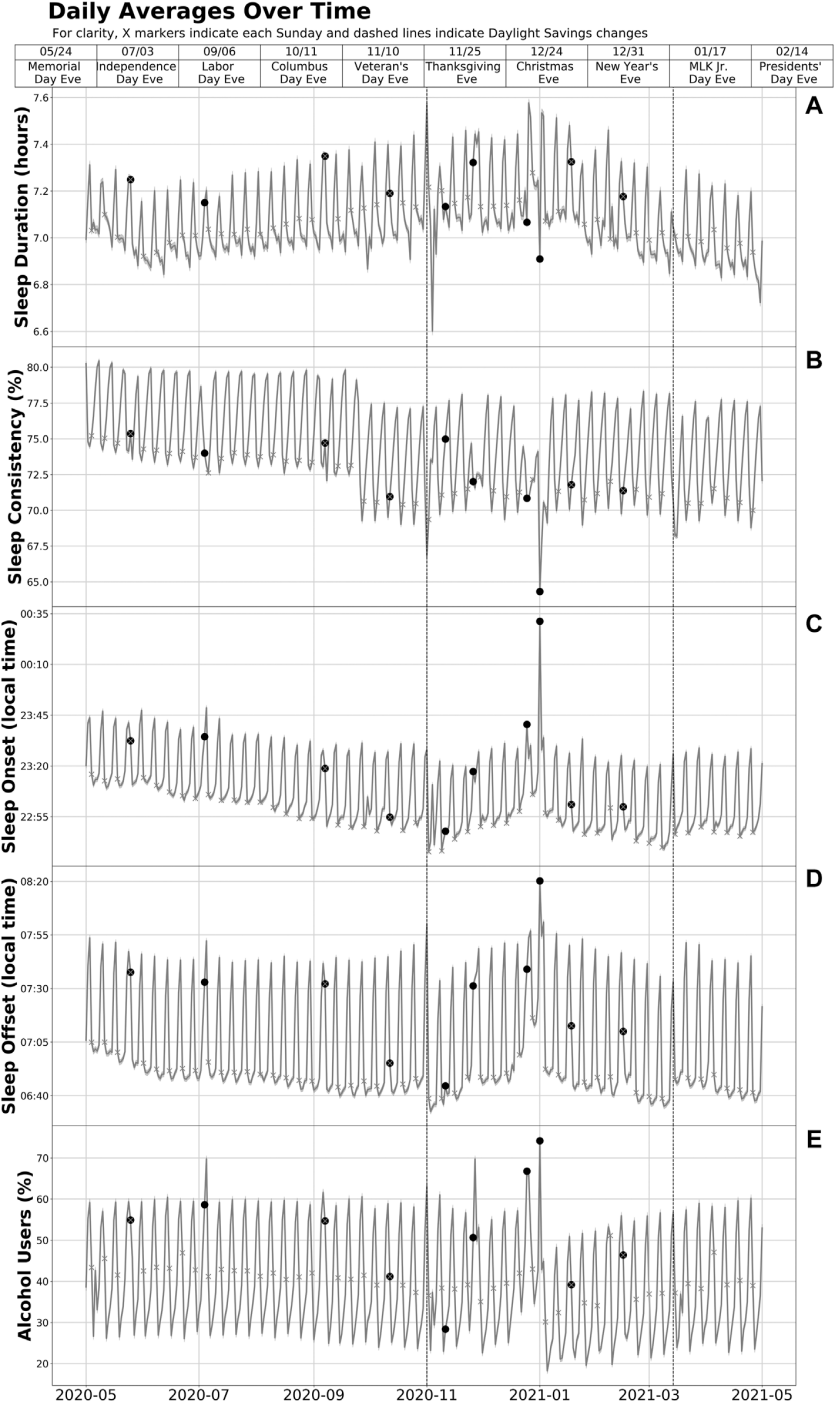
Sleep duration **(A)**, consistency **(B)**, timing **(C, D)**, and alcohol use **(E)** among US Adult WHOOP Users During 1 May 2020—1 May 2021. Data were collected on participants’ sleep duration, sleep consistency, sleep timing, and alcohol use from 1 May 2020 through 1 May 2021. This figure shows the daily sample means and 95% confidence intervals over the study interval for sleep duration, consistency, and timing based on data from the 10,350,760 sleep episodes recorded by the 24,250 participants in the primary analytic sample. The measure for alcohol is the percentage of users who responded affirmatively to having used alcohol in daily surveys among the 13,904 participants in the subsample with data on alcohol use. Each round scatter point represents the eve of a US public holiday or DST transition and indicates a nocturnal sleep episode that ended the following day. For example, the point on 31/12/20 (New Year’s Eve) represents the nocturnal sleep episode that took place from the evening of 31 December 2020 (or the early morning hours of 1 January 2021) through the morning of 1 January 2021.

Holidays, their eves, and DST transitions represented several local minima and maxima in sleep and alcohol data [with the US Presidential Election (3 November 2020) also associated with marked changes in sleep and alcohol use] (Figure 1). The holidays and their eves also stand apart from surrounding weekends, suggesting a potentially independent effect beyond that of a standard day off from work. Nearly all holidays were significantly different from baseline in each of the assessed outcomes.

### Sleep Duration (Figure 2A)

The mean absolute change (average percent change in absolute value) from baseline sleep duration during US public holidays during the study interval was 1.9%. Including holiday eves, the absolute change was 2.1%. Overall, 15 of the 20 (75%) holidays and eves were associated with significant increases in average sleep duration, with an average increase of 2.5% (Table 1). There were significant decreases in sleep duration on two of the 20 (10%) holidays and eves, with an average decrease of 1.4%. Increases and decreases in average sleep duration were observed following the Fall Back and Spring Ahead DST transitions, respectively. The largest average increases from baseline sleep duration occurred on Thanksgiving Day [5.2% ± 0.3 (7h31 m vs*.* 7h10 m)], Christmas Day [5.1% ± 0.3 (7h38 m vs*.* 7h18 m)], the Fall Back DST transition [4.9% ± 0.3 (7h39 m vs*.* 7h19 m)], and New Year’s Day [4.8% ± 0.3 (7h37 m vs*.* 7h17 m)]. The largest average decreases from baseline sleep duration occurred on New Year’s Eve [−2.5% ± 0.3 (6h58 m vs*.* 7h10 m)] and the Spring Ahead DST transition [−0.8% ± 0.3 (7h07 m vs*.* 7h12 m)], with Memorial Day [−0.3% ± 0.2 (7h03 m vs*.* 7h05 m)] as the only other public holiday with a significant decrease in sleep duration. Changes to sleep duration on Christmas Eve, Columbus Day, and Veterans Day were not significant (*p* > 0.05) (Tables 1, 2).

**FIGURE 2:**
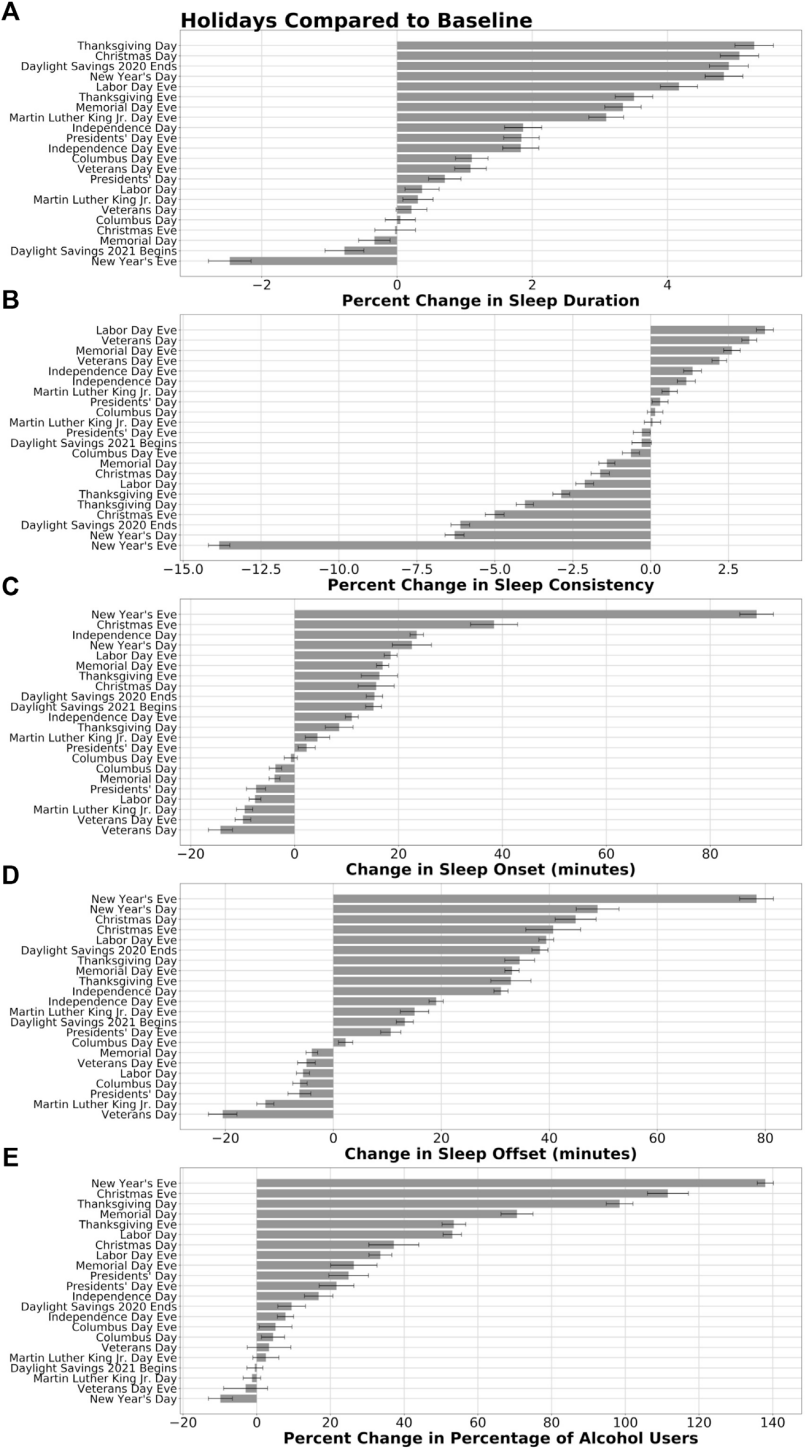
Sleep and alcohol use during DST transitions and on holidays and their eves compared to baseline. The percent change from baseline sleep duration **(A)** and sleep consistency **(B)**, change in minutes of sleep onset and offset timing **(C, D)**, and percent change in the percentage of participants reporting alcohol use **(E)** were calculated across the sample for DST transitions and each of the US public holidays and eves are shown. For each measure, holidays, eves, and DST transitions were ranked from the largest increase to the largest decrease compared to baseline sleep and alcohol use measures. Error bars on sleep duration, consistency, and timing panels represent 95% confidence intervals. Error bars on the alcohol panel represent the propagation of error from binomial proportion confidence intervals of the holidays and their baselines.

**TABLE 1:** Percent change to sleep and alcohol use patterns during DST transitions and on holidays and their eves compared to baseline.

	Sleep duration in hrs (n = 24,250)		Sleep consistency (0–100) (n = 24,250)		Sleep onset (hh:mm) (n = 24,250)		Sleep offset (hh:mm) (n = 24,250)		Alcohol use (% of users) (n = 13,904)	
Holiday	Mean	Error	Mean	Error	Mean	Error	Mean	Error	Mean	Error
Memorial Day Eve	3.339	0.269	2.611	0.265	16.95	1.18	33.10	1.133	26.35	3.255
Memorial Day	−0.336	0.232	1.403	0.256	−3.87	1.04	−3.98	1.08	70.62	5.95
Independence Day Eve	1.830	0.267	1.346	0.285	11.02	1.23	19.04	1.31	7.80	2.37
Independence Day	1.864	0.275	1.150	0.287	23.52	1.29	31.06	1.31	16.82	2.16
Labor Day Eve	4.165	0.275	3.668	0.273	18.51	1.24	39.43	1.36	33.54	3.54
Labor Day	0.363	0.252	−2.112	0.286	−7.66	1.13	−5.73	1.22	53.10	5.90
Columbus Day Eve	1.083	0.241	−0.597	0.274	−0.89	1.26	1.95	1.32	5.12	3.16
Columbus Day	0.021	0.222	0.171	0.249	−3.74	1.20	−6.74	1.32	4.43	4.47
Fall Back DST Transition	4.907	0.287	−6.104	0.298	15.36	1.58	38.27	2.77	9.47	0.85
Veterans Day Eve	1.048	0.234	2.258	0.236	−10.50	1.48	−5.92	1.61	−3.03	3.76
Veterans Day	0.191	0.227	3.198	0.239	−14.75	2.26	−21.24	2.57	3.34	3.87
Thanksgiving Eve	3.451	0.227	−2.812	0.269	15.20	3.26	30.85	3.60	53.48	4.72
Thanksgiving Day	5.245	0.284	−3.989	0.277	8.33	2.60	34.09	2.72	98.44	5.37
Christmas Eve	−0.034	0.301	−5.001	0.301	38.08	4.49	40.33	5.00	111.56	6.23
Christmas Day	5.059	0.283	−1.616	0.294	15.67	3.47	44.75	3.76	37.18	3.10
New Year’s Eve	−2.476	−0.316	−13.844	0.345	88.87	3.22	78.13	3.13	137.98	6.80
New Year’s Day	4.831	0.278	−6.284	0.306	22.57	3.74	48.67	3.93	−9.86	2.48
Martin Luther King Jr. Day Eve	3.092	0.257	0.064	0.263	4.04	2.41	15.01	2.61	2.48	3.21
Martin Luther King Jr. Day	0.312	0.224	0.618	0.247	−9.64	1.56	−12.48	1.59	−1.28	4.33
Presidents’ Day Eve	1.836	0.262	−0.269	0.273	1.94	1.61	10.51	2.86	21.67	3.61
Presidents’ Day	0.710	0.240	0.328	0.255	−8.00	178	−6.60	2.60	24.93	5.56
Spring Ahead DST Transition	−0.777	0.286	−0.288	0.313	15.19	1.53	13.24	1.58	−0.52	0.89

**TABLE 2:** Sleep and alcohol use patterns at baseline and during DST transitions and on holidays and their eves.

	Sleep duration in hrs (n = 24,250)		Sleep consistency (0–100) (n = 24,250)		Sleep onset (hh:mm) (n = 24,250)		Sleep offset (hh:mm) (n = 24,250)		Alcohol use (% of users) (n = 13,904)	
Holiday	Mean	Error	Mean	Error	Mean	Error	Mean	Error	Mean	Error
	Baseline	Baseline	Baseline	Baseline	Baseline	Baseline	Baseline	Baseline	Baseline	Baseline
	Holiday	Holiday	Holiday	Holiday	Holiday	Holiday	Holiday	Holiday	Holiday	Holiday
Memorial Day Eve	7h06 m	0.7 h	74.5	0.1	22:41	1 m	06:37	1 m	43.4%	0.9%
	7h19 m	1.2 h	75.9	0.2	23:04	1 m	07:14	2 m	54.9%	0.9%
Memorial Day	7h05 m	0.7 h	75.4	0.1	22:37	1 m	06:31	1 m	26.7%	0.8%
	7h03 m	1.1 h	74.1	0.2	22:41	1 m	06:32	1 m	45.5%	0.9%
Independence Day Eve	7h06 m	0.7 h	74.0	0.1	22:48	1 m	06:45	1 m	54.4%	0.9%
	7h13 m	1.2 h	74.5	0.2	23:05	1 m	07:08	2 m	58.6%	0.9%
Independence Day	7h10 m	0.7 h	73.4	0.1	22:52	1 m	06:53	1 m	59.7%	0.9%
	7h16 m	1.2 h	73.7	0.2	23:19	1 m	07:28	2 m	69.7%	0.8%
Labor Day Eve	7h08 m	0.7 h	73.1	0.1	22:25	1 m	06:23	1 m	40.9%	0.9%
	7h24 m	1.1 h	75.3	0.2	22:49	1 m	07:07	1 m	54.7%	0.9%
Labor Day	7h06 m	0.7 h	74.1	0.1	22:24	1 m	06:20	1 m	25.6%	0.8%
	7h06 m	1.2 h	72.3	0.2	22:22	1 m	06:18	2 m	39.2%	0.9%
Columbus Day Eve	7h11 m	0.7 h	72.3	0.1	22:18	1 m	06:21	1 m	39.2%	0.8%
	7h15 m	1.1 h	71.5	0.2	22:25	1 m	06:29	1 m	41.2%	0.9%
Columbus Day	7h08 m	0.7 h	73.4	0.1	22:18	1 m	06:17	1 m	24.1%	0.7%
	7h07 m	1.1 h	73.2	0.2	22:21	1 m	06:16	1 m	25.2%	0.8%
Fall Back DST Transition	7h19 m	0.7 h	71.9	0.1	22:37	1 m	06:52	1 m	57.8%	0.9%
	7h39 m	1.3 h	67.3	0.2	22:58	2 m	07:35	2 m	63.2%	0.9%
Veterans Day Eve	7h08 m	0.7 h	74.2	0.1	22:20	2 m	06:21	2 m	29.3%	0.8%
	7h11 m	1.1 h	75.5	0.2	22:18	1 m	06:19	1 m	28.4%	0.8%
Veterans Day	7h10 m	0.7 h	75.0	0.1	22:38	3 m	06:38	3 m	29.9%	0.8%
	7h09 m	1.1 h	77.0	0.2	22:17	1 m	06:15	2 m	30.9%	0.8%
Thanksgiving Eve	7h11 m	0.7 h	75.2	0.1	22:35	3 m	06:36	3 m	33.0%	0.8%
	7h24 m	1.2 h	72.7	0.2	22:46	2 m	07:06	2 m	50.7%	0.9%
Thanksgiving Day	7h10 m	0.7 h	75.7	0.1	22:30	3 m	06:34	2 m	35.1%	0.9%
	7h31 m	1.2 h	72.3	0.2	22:40	2 m	07:10	2 m	69.7%	0.8%
Christmas Eve	7h11 m	0.8 h	75.7	0.2	22:41	5 m	06:45	4 m	31.6%	0.8%
	7h09 m	1.3 h	71.4	0.2	23:10	2 m	07:15	2 m	66.8%	0.9%
Christmas Day	7h18 m	0.8 h	73.2	0.1	22:40	3 m	06:45	3 m	48.0%	0.9%
	7h38 m	1.2 h	71.5	0.2	22:53	2 m	07:30	2 m	65.9%	0.8%
New Year’s Eve	7h11 m	0.7 h	75.8	0.1	22:33	3 m	06:39	2 m	31.2%	0.8%
	6h58 m	1.4 h	65.0	0.3	00:01	2 m	07:56	2 m	74.2%	0.8%
New Year’s Day	7h17 m	0.7 h	73.2	0.1	22:38	4 m	06:44	3 m	47.5%	0.9%
	7h37 m	1.3 h	68.3	0.2	22:57	2 m	07:31	2 m	42.9%	0.9%
Martin Luther King Jr. Day Eve	7h11 m	0.7 h	72.6	0.1	22:29	2 m	06:36	2 m	38.2%	0.9%
	7h23 m	1.2 h	72.3	0.2	22:32	1 m	06:48	2 m	39.2%	0.9%
Martin Luther King Jr. Day	7h10 m	0.7 h	73.2	0.1	22:24	2 m	06:29	2 m	24.2%	0.7%
	7h11 m	1.1 h	73.5	0.2	22:18	1 m	06:19	1 m	23.8%	0.7%
Presidents’ Day Eve	7h07 m	0.7 h	72.5	0.1	22:26	2 m	06:33	2 m	38.1%	0.9%
	7h14 m	1.2 h	75.0	0.2	22:29	2 m	06:44	2 m	46.4%	0.9%
Presidents’ Day	7h06 m	0.7 h	73.4	0.1	22:28	2 m	06:32	2 m	21.7%	0.7%
	7h07 m	1.1 h	73.4	0.2	22:18	1 m	06:25	1 m	27.2%	0.8%
Spring Ahead DST Transition	7h12 m	0.7 h	72.0	0.2	22:34	1 m	06:51	1 m	56.9%	0.9%
	7h05 m	1.2 h	71.5	0.2	22:54	2 m	07:08	2 m	56.7%	0.9%

### Sleep Consistency (Figure 2B)

The mean absolute change from baseline sleep consistency during US public holidays was 2.1%. Including eves, the absolute change was 2.8%. The eight holidays with significant increases in sleep consistency had an average increase of 1.9%. Nine of 20 (45%) holidays and eves were associated with significant decreases in sleep consistency, with an average decrease of 4.2%. New Year’s Eve had the largest change [−13.8% ± 0.3 (65.0 vs*.* 75.8 out of 100)] (Table 1). The next largest changes were observed on New Year’s Day [−6.3% ± 0.3 (68.3 vs*.* 73.2)], the Fall Back DST transition [−6.1% ± 0.3 (67.3 vs*.* 71.9)], and Christmas Eve [−5.0% ± 0.3 (71.5 vs*.* 75.7)]. The night before Labor Day had the highest average increase in sleep consistency from baseline [2.6% (75.3 vs*.* 73.1)]. Sleep consistency differences spanned a larger range (20.2%) when compared to sleep duration differences (16.5%). The percent changes in sleep consistency on Columbus Day, the night before Martin Luther King Jr Day, and the Spring Ahead DST transition were not significant (*p* > 0.05) (Tables 1, 2).

### Sleep Onset (Figure 2C)

The mean absolute change from baseline in the timing of sleep onset was 11.8 min on public holidays. Including their eves, sleep onset changed on average 16.2 min. Most changes in sleep onset timing [12 of 20 (60%)] were delays in sleep onset, indicating a population-level bedtime that was, on average, 22.1 min later on those holidays (Table 1). The largest average delay in sleep onset was on New Year’s Eve, on which the average change was an 88.9-min (±3.2) delay in sleep onset time, from a baseline average of 22:33 to 00:01 on New Year’s Eve (Table 2). Later sleep onset times were also observed with Spring Ahead and Fall Back DST transitions. Seven of the 20 (35%) holidays and eves were associated with significantly earlier sleep onset times compared to baseline—on average 8.3 min earlier (Table 1). Each of the holidays that had an earlier average time of sleep onset, indicating an earlier average bedtime, were on Monday holidays.

### Sleep Offset (Figure 2D)

The mean absolute change from baseline in the timing of sleep offset on public holidays was 21.5 min. This was almost twice the average change of sleep onset timing. Including the holiday eves, the average change was 24.5 min 13 of 20 (65%) holidays and eves were associated with a later sleep offset—on average 32.8 min later. Both Spring Ahead and Fall Back DST transitions were associated with delayed sleep offset as well, even following the Fall Back DST transition when an extra hour was added to the night. On New Year’s Eve (the morning of New Year’s Day), offset timing delayed 78.1 min (±3.1) later—from a 06:39 baseline to 07:56 on the morning after New Year’s Eve—the largest change in sleep offset (Table 1). We observed earlier sleep offsets (on average 8.9 min earlier) on seven of the 20 (35%) holidays and eves, each of which were Monday holidays (Table 1).

### Alcohol Use (Figure 2E)

The mean absolute change from the baseline alcohol use on holidays was 32.0%. Including their eves, the mean absolute change was 36.8%. We observed a significantly increased prevalence of alcohol consumption on 14 of 20 (70%) holidays and eves, with an average increase of 46.7% (Table 1). The majority of significant changes in the number of alcohol consumers were increases, with one exception of New Year’s Day, when alcohol consumption averaged 9.9% lower than surrounding same days of the week. More than twice as many users reported alcohol consumption on New Year’s Eve [138.0% ± 6.7 (74.2% vs*.* 31.2%)] and Christmas Eve [111.6% ± 4.7 (66.7% vs*.* 31.6%)] compared to the baseline (Tables 1 and 2). Regarding DST transitions, the point prevalence of alcohol use increased by 9.5% ± 0.85 during the Fall Back DST transition (63.2% vs*.* 57.7%), and did not differ significantly during the Spring Ahead DST transition (56.7% vs*.* 56.9%).

### Paired Alcohol Use and (1) Sleep Duration and (2) Sleep Consistency (Figure 3)

**FIGURE 3:**
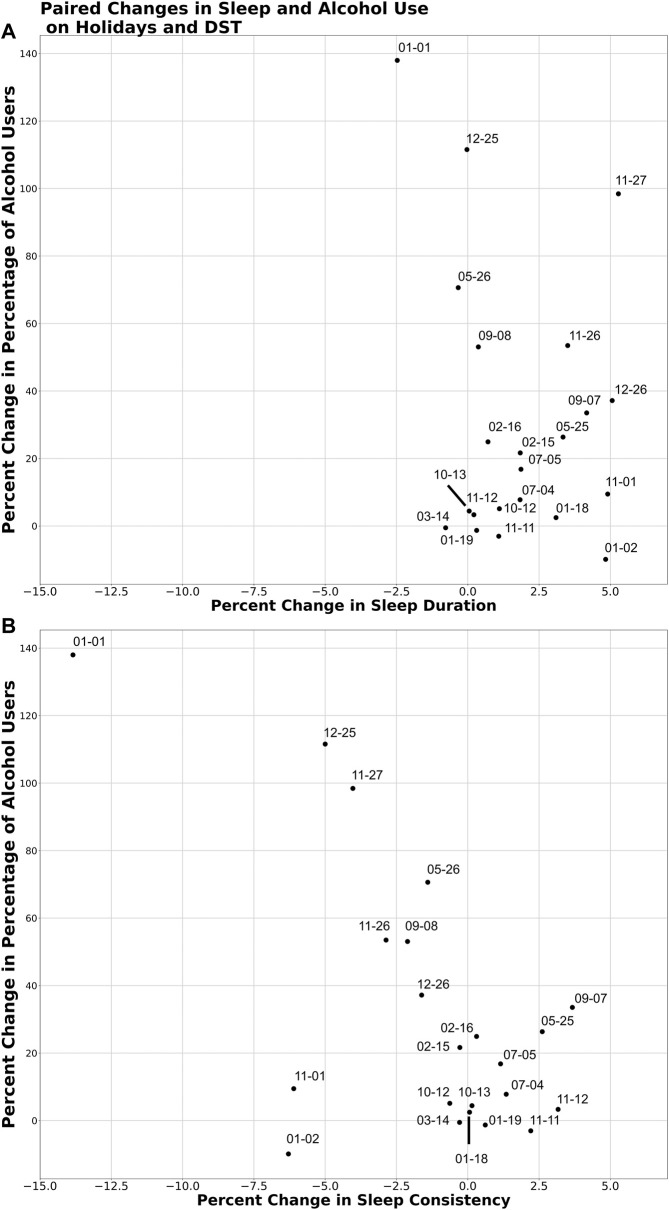
Paired changes to alcohol use and **(A)** sleep duration and **(B)** sleep consistency during DST transitions and on holidays and their eves compared to baseline. The percent change in the point prevalence of alcohol use and each of sleep duration and sleep consistency were plotted. Correlation coefficients were calculated to estimate the linear relationship between each of these paired variables.

The analysis of paired alcohol use and each of sleep duration and sleep consistency revealed a non-significant correlation (*r* = −0.211; *p* = 0.34) between changes in alcohol use and changes in sleep duration (Figure 3A), yet a strong, negative correlation (*r* = −0.635; *p* = 0.001) between alcohol use and sleep consistency, with an increased percentage of alcohol use associated with decreased sleep consistency across the sample (Figure 3B).

## Discussion

We found that sleep and alcohol use were significantly different from baseline on the majority of US public holidays and their preceding days, as well as during DST transitions, among a large (*n* = 24,250) US-based sample of adults aged 18–65 years who were regular users of the wearable biometric device platform WHOOP. These objective data from year-long recordings using a regularly worn validated device reveal for the first time that US public holidays are associated with significant changes in sleep duration, consistency, and timing compared to baseline sleep patterns. Overall, we found that most holidays and their eves were associated with increased sleep duration, decreased sleep consistency, later bedtimes and wake times (with the exception of Monday holidays), and increased prevalence of alcohol use. The Fall Back DST transition was associated with an increase in sleep duration and decrease in consistency, whereas the Spring Ahead DST transition was associated with a decrease in sleep duration and no significant difference in sleep consistency. Given the high-magnitude changes observed on some holidays, the health implications of marked, acute alterations to sleep and alcohol use patterns on holidays compared with baseline behaviors warrant further investigation.

Holidays that frequented the highest magnitude changes in sleep and alcohol use patterns included New Year’s Eve, New Year’s Day, Christmas Eve, Christmas Day, and Thanksgiving Day. Indeed, the largest average increase from baseline sleep duration occurred on Thanksgiving Day (5.2% ± 0.3), with participants exhibiting a larger percentage increase in sleep duration than that observed on the Fall Back DST transition (a night in which an “extra hour” of sleep is afforded by resetting the clocks an hour earlier). New Year’s Eve was associated with the largest change in sleep consistency, a decrease of 13.8% ± 0.3, and the largest change in sleep onset, which occurred an average of 88.9 ± 3.2 min later. Participants also had an average sleep offset 78.1 ± 3.1 min later on New Year’s Eve (the morning of New Year’s Day). More than twice as many participants reported alcohol consumption on New Year’s Eve (138.0% ± 6.7) and Christmas Eve (111.6% ± 4.7) when compared to baseline. Notably, the largest decrease in alcohol consumption prevalence relative to baseline also occurred on New Year’s Day, 1 day after the largest increase in point prevalence of alcohol use.

Analysis of paired changes in the prevalence of alcohol use and 1) sleep duration and 2) sleep consistency revealed that while there was not a strong relationship between alcohol use and sleep duration, increased prevalence of alcohol use correlated with decreased consistency of sleep timing. As a central nervous system depressant, alcohol consumption can lead to decreased sleep latency (Colrain et al., 2014). However, high blood alcohol levels are also associated with alterations in sleep architecture and disrupted, poor quality sleep—with more nighttime awakenings—throughout the night. While the absence of morning commitments on holidays might compensate for reduced sleep efficiency in terms of attaining sufficient sleep duration, the quality of the sleep might be impaired. The direction of the relationship between increased prevalence of alcohol use and decreased sleep consistency was not assessed in this analysis, and could be due to multiple factors, including the variables assessed and holiday-related social jet lag (e.g., staying up later to drink socially). In the case of social events, decreased sleep consistency might be observed owing to socializing at night, regardless of whether or not alcohol were involved.

Additional research on the public health effects of such marked, acute changes in sleep patterns and alcohol use would help to elucidate the implications of a combination of changes that have been associated with improvements to health (e.g., increased sleep duration) and challenges to health (e.g., delayed sleep timing, increased alcohol use). For example, as described in the Introduction, DST transitions have been researched extensively, providing evidence that the Spring Ahead DST transition is associated with increased motor vehicle crashes, workplace injuries, heart attacks, and all-cause mortality (Barnes and Wagner, 2009; Sandhu et al., 2014; Manfredini et al., 2019; Fritz et al., 2020; Poteser and Moshammer, 2020). Holidays do not present the same acute perturbation of the timing of environmental light-dark cycles as DST transitions, though comparatively larger changes to sleep and alcohol use patterns were observed on several holidays (especially the days of and before Thanksgiving, Christmas, and New Year’s), suggesting the need for future research to investigate the impact of these changes on health.

Moreover, many of the holidays and eves were associated with increases in sleep duration larger than the effect of melatonin on patients with sleep disorders [+8.25 min (95% CI 1.74 to 14.75), *p* = 0.013] (Ferracioli-Oda et al., 2013) and the impact of Cognitive Behavioral Therapy for Insomnia (CBT-I) [+7.61 min (95% CI -0.51 to 15.74)] (Trauer et al., 2015). While melatonin and CBT-I are designed for people with sleep conditions and the study sample did not assess for diagnosed sleep conditions, the comparison highlights the magnitude of changes to sleep patterns observed on holidays and their eves.

Of note, the Fall Back DST transition occurred at 02:00 on 1 November 2020, with the widely recognized and nocturnally celebrated Halloween occurring on 31 October 2020. The holiday-like nature of Halloween might have influenced the observations on the Fall Back DST transition, including a higher prevalence of alcohol use and delayed sleep onset and offset (despite the clocks moving 1 hour earlier at 02:00 on the morning of the Fall Back DST transition). Future investigations should explore the extent to which these observations exist when the Fall Back DST transition does not overlap with Halloween.

### Strengths and Limitations

Strengths of this study include the large sample size and the utilization of sleep-wake data that were 1) high-resolution (i.e., continuous 1 Hz recordings to generate 30-s epoch resolution sleep stage estimates) 2) largely complete (i.e., minimal missingness, with data for an average of 87.9% of the study interval), and 3) comprehensive (i.e., capturing multiple dimensions of sleep patterns, including the duration and timing of sleep), collected from a sleep wearable device validated against polysomnography (Berryhill et al., 2020). Additionally, in the alcohol use subsample, daily assessment of the variable minimized potential recall bias.

Limitations of this study may include selection bias potentially amplified by the Big Data Paradox, self-reported alcohol data, and potential unmeasured effects of the COVID-19 pandemic. Regarding selection bias, the data were retrospectively analyzed from subscribers to the WHOOP platform. Given that WHOOP is a personalized digital fitness and health platform, participants may be more informed about sleep and its impact on performance compared to the general population. Moreover, demographic characteristics outside of sex and age were not collected, so beyond the overrepresentation of men in the study sample, the extent to which the sample is representative of the US adult population is unknown. Sample under-representativeness can be especially concerning with large datasets such as the one used for this analysis, as potentially incorrect point estimates of biased samples can be compounded by substantially underestimated widths of confidence intervals afforded by large sample sizes—the Big Data Paradox (Meng, 2018; Bradley et al., 2021). Regarding self-reported alcohol data, surveys could be subject to social desirability and other biases. For alcohol data in particular, as survey responses were opt-in for subscribers; subscribers who rarely drink alcohol may have been less likely to track their alcohol use. The data shown should be interpreted as relative drinking prevalence among those who drink, which may overrepresent the true prevalence of population-level drinking. On the other hand, if the findings in our sample were generalizable, the increased prevalence on holidays may underestimate the population-level increase. Finally, the study interval analyzed occurred entirely during the COVID-19 pandemic, and the associated stay-at-home orders and social distancing mandates may have impacted the behavioral changes ordinarily associated with holiday observance. However, the selection of the study interval as beginning on 1 May 2020 was partially to avoid the interval during which pandemic effects on population movement would be strongest. Mobility data reveal that population movement increased rapidly following the lifting of the first stay-at-home order, which occurred prior to the start of the study interval (24 April 2020), suggesting that the peak impact of mobility restrictions due to the pandemic had passed (Moreland et al., 2020). Nevertheless, further research following the complete lifting of social distancing mandates will be required to understand how these data were impacted by the present mandates.

The results of these analyses should be considered with the understanding that a large data set with nonrandom recruitment was used. As such, due to the nature of WHOOP platform membership at the time of the study, most participants were male. The big data paradox is minimized in this analysis given the use of real-world data to assess discrete hypotheses and an analysis plan that was determined before beginning research and carried through to completion. Although estimating expected systematic differences in outcomes with a more representative sample is extremely difficult, we found no significant differences to report when stratifying the presented analyses by age and sex groups. Additionally, results could be somewhat confounded by behavior changes of participants due to COVID-19 pandemic as the time interval analyzed coincided with an active interval of COVID-19. This could lead to an underestimation of the impact of holidays as there were later bed times and wake times and longer sleep duration overall during this interval (Robbins et al., 2021). Sleep consistency had also increased during the pandemic (Czeisler et al., 2022) potentially leading to an overestimation in the changes in sleep consistency associated with holidays.

## Conclusion

Overall, in this non-random sample of US adult subscribers to a personalized digital fitness and health platform, we found that most US public holidays and their eves, as well as DST transitions, were associated with significant changes in sleep duration, sleep consistency, and sleep timing among a large sample of wearers of a commercial fitness tracker. Holidays—more than DST transitions—were also associated with changes in the point prevalence of alcohol use. Similar magnitude changes to sleep and alcohol consumption have been associated with adverse health impacts. As such, this research warrants further investigation of the behavioral changes associated with public holidays.

## Data Availability

The datasets presented in this article are not readily available because Public data sharing for these purposes is ethically and legally restricted. The data is collected and used with the consent of individuals who purchase a WHOOP membership (collectively, “WHOOP Members”) and agree to the WHOOP terms of use (https://www.whoop.com/termsofuse/) and privacy policy (https://www.whoop.com/privacy/full-privacy-policy/). While WHOOP terms of use and privacy policy permit WHOOP to use collected data that has been aggregated or de-identified in a manner that no longer identifies an individual for informational purposes, analytics or WHOOP’s own research purposes, the consent obtained from WHOOP Members does not extend to making the data publicly available for a third party to use for its own purposes. As such, WHOOP’s legal department will not permit the data to be shared for these purposes. Data access queries can be directed to the corresponding author. Requests to access the datasets should be directed to emily@whoop.com.
